# Survival endpoints in colorectal cancer and the effect of second primary other cancer on disease free survival

**DOI:** 10.1186/1471-2407-11-438

**Published:** 2011-10-11

**Authors:** Helgi Birgisson, Ulrik Wallin, Lars Holmberg, Bengt Glimelius

**Affiliations:** 1 Department of Surgical Sciences, Colorectal Surgery, Uppsala University, Uppsala, Sweden; 2 Division of Colon and Rectal Surgery, Department of Surgery, University of Minnesota, USA; 3 King's College London, Division of Cancer Studies Cancer Epidemiology Group, London, UK; 4 Department of Radiology, Oncology, and Radiation Science, Uppsala University, Uppsala, Sweden; 5 Department of Oncology and Pathology, Karolinska Institutet, Stockholm, Sweden

## Abstract

**Background:**

In cancer research the selection and definitions of survival endpoints are important and yet they are not used consistently. The aim of this study was to compare different survival endpoints in patients with primary colorectal cancer (CRC) and to understand the effect of second primary other cancer on disease-free survival (DFS) calculations.

**Methods:**

A population-based cohort of 415 patients with CRC, 332 of whom were treated with curative intention between the years 2000-2003, was analysed. Events such as locoregional recurrence, distant metastases, second primary cancers, death, cause of death and loss to follow-up were recorded. Different survival endpoints, including DFS, overall survival, cancer-specific survival, relapse-free survival, time to treatment failure and time to recurrence were compared and DFS was calculated with and without inclusion of second primary other cancers.

**Results:**

The events that occurred most often in patients treated with curative intention were non-cancer-related death (n = 74), distant metastases (n = 66) and death from CRC (n = 59). DFS was the survival endpoint with most events (n = 170) followed by overall survival (n = 144) and relapse-free survival (n = 139). Fewer events were seen for time to treatment failure (n = 80), time to recurrence (n = 68) and cancer-specific survival (n = 59). Second primary other cancer occurred in 26 patients and its inclusion as an event in DFS calculations had a detrimental effect on the survival. The DFS for patients with stage I-III disease was 62% after 5 years if second primary other cancer was not included as an event, compared with 58% if it was. However, the difference was larger for stage II (68 vs 60%) than for stage III (49 vs 47%).

**Conclusions:**

The inclusion of second primary other cancer as an endpoint in DFS analyses significantly alters the DFS for patients with CRC. Researchers and journals must clearly define survival endpoints in all trial protocols and published manuscripts.

## Background

In cancer research the selection and definition of survival endpoints are important and among the central issues discussed in the Consolidated Standards of Reporting Trials (CONSORT) statement [1,2]. The definitions of the endpoints are often missing in clinical trials, even in the majority of randomised controlled cancer trials [3] making it difficult to compare studies. Meta-analyses will not be reliable when studies with different endpoint definitions are being compared.

It is central in the quest for improved cancer treatment that we minimize problems related to study analyses. One way to do this is always to use endpoints that have the same definitions.

There are several difficulties related to the present use of endpoints. The nomenclature is not straightforward as it is not always clear from the name of the survival endpoint what events it includes. It is also problematic to have an event such as second primary cancer that is frequently encountered but inconsistently included in the endpoints. It was therefore an excellent initiative to publish a consensus report on the definitions of endpoints used in adjuvant treatment trials in CRC (Table 1) [4].

**Table 1:** Definition of endpoints and events as suggested by the consensus agreement of Punt et al. J Natl Cancer Inst 2007; 99(13): 998-1003.

		Endpoint				
**Event**	**DFS**	**RFS**	**TTR**	**TTF**	**CSS**	**OS**

Locoregional recurrence	E	E	E	E	I	I

Distant metastases	E	E	E	E	I	I

Second primary, same cancer	E	I	I	E	I	I

Second primary, other cancer	E	I	I	E	I	I

Death from same cancer	E	E	E	E	E	E

Death from other cancer	E	E	C	E	C	E

Non - cancer-related death	E	E	C	C	C	E

Treatment-related death	E	E	C	E	C	E

Loss to follow-up	C	C	C	C	C	C

DFS = disease-free survival; RFS = relapse-free survival; TTR = time to recurrence; TTF = time to treatment failure; CSS = cancer specific survival; OS = overall survival; E = event; C = censor; I = ignore

The selection of survival endpoints depends on the study question and the information available. It is important to predefine the endpoints that should be collected during the study design phase since prospectively sampled information is more reliable and complete than information sampled retrospectively. Second primary other cancer is probably more often thought of as a late adverse effect or a competing risk than as a part of a survival endpoint. It can therefore be forgotten in treatment trials when the treatment is not thought to induce second primary other cancers and may not be available for the survival calculations. The use of disease-free survival (DFS) rather than overall survival (OS) has become more frequent in adjuvant cancer trials [5]. DFS offers earlier presentation of data as events due to disease recurrence by nature occur earlier than death from the disease. Besides, there are more events in DFS than in OS, as events, such as disease recurrence, and second primary other cancers that do not necessarily lead to death are included in DFS but not in OS. Despite the increased use of DFS as an endpoint, its definition varies widely between studies, in particular regarding the occurrence of a second primary other cancer [6]. Research on how DFS is affected by the inclusion or exclusion of second primary other cancers as an endpoint in DFS calculations is lacking. Therefore this study was initiated using a population-based cohort with well-documented follow-up data including information on second primary other cancers.

The aim was to compare different survival endpoints in patients with primary CRC according to the work by Punt et al (Table 1) and to better understand the relevance of inclusion or exclusion of second primary other cancers as an endpoint in the DFS calculations.

## Methods

The study cohort included 415 patients consecutively diagnosed and treated for CRC in the county of Västmanland, Sweden, which has a population of 260 000 inhabitants. The time period of diagnosis was between August 2000 and December 2003. The patients were identified through the Regional Cancer Registry which forms the regional report to the Swedish Cancer Registry, to which the reporting is mandated by law and which has a high completeness [7,8]. It is estimated that more than 99% of all incident colorectal cancers in the county are included in the cohort. The majority of the patients were treated at the Central District Hospital in Västerås. During the time-period for this study patients undergoing surgery for CRC were invited to donate tumour tissue and blood for future studies (n = 322) [9]. Information on family history was prospectively collected from 318 patients [10]. The information on tumour stage and grade of tumour differentiation was gained from pathology reports. Information on second cancer, cancer recurrence, death and causes of death was obtained by matching with the Clinical Database for Colorectal Cancer held at the Regional Oncologic Center in the Uppsala/Örebro region [11] and from hospital records at the Departments of Surgery, Oncology and Pathology at the hospital in Västerås.

Treatment-related death and date of death were available for both colon and rectal cancer patients, but information on locoregional recurrence and distant metastases was available only for rectal cancer patients. This information was double-checked during retrieval of data from hospital records. Other survival endpoints not available in the clinical database were retrieved from hospital records. If patients had moved from the county of Västmanland to another part of Sweden copies of their hospital records were obtained from their local hospital. One patient was lost to follow-up due to emigration.

Of the 415 patients, the following were omitted from the analyses: 68 patients with metastatic disease at presentation, 4 patients with stage II and III disease that were non-radically operated, and 11 patients with unknown disease stage of whom 7 were not operated. Remaining for participation in the analyses were 332 patients treated with curative intention with complete information on disease stage. Four patients treated with local excision of T1 tumours and polyp cancers were classified as curatively treated. Although the screening for metastases proved negative for the four patients treated with local excision, their lymph node status and, hence, disease stages were unknown.

The guidelines for observational studies in epidemiology (STROBE) were followed during the preparation of the manuscript [12]. Ethical approval was obtained from the Ethics committee at Uppsala University, Uppsala, Sweden.

### Statistical methods

Endpoints were defined according to Punt et al (Table 1) [4]. All observations were censored at loss to follow-up and at the end of the study period (April 15 2010). OS was measured from the date of surgery to the date of death from any cause; locoregional recurrences, distant metastases and second primary cancer were ignored. DFS was measured from the date of surgery to the date of second cancer, locoregional recurrence, distant metastases or death from any cause. Cancer-specific survival (CSS) was measured from the date of surgery to the date of death from CRC; the observations were censored at death from causes other than CRC; locoregional recurrences, distant metastases and second primary cancer were ignored. Time to recurrence (TTR) was measured from the date of surgery to the date of locoregional recurrence, distant metastases or to the date of death from CRC; the observations were censored at the date of death in non-CRC and second primary cancer was ignored. Relapse-free survival (RFS) was measured from the date of surgery to the date of recurrence or death from any cause; second primary cancer was ignored. Time to treatment failure (TTF) was measured from the date of surgery to the date of second cancer, locoregional recurrence, distant metastases or death from cancer and treatment-related death; patients were censored at non-cancer related death.

OS and CSS were calculated for all patients and DFS, TTR, TTF and RFS were calculated in patients treated with curative intention defined as having disease stage I-III with both macro- and microscopic free resection margins (R0).

Survival curves for all endpoints were plotted to better understand where endpoints stood in relation to one another in this cohort. The Kaplan Meier method was used to calculate the cumulative proportion surviving and to plot the survival curves. The Mann-Whitney U test was used in comparisons of non-parametric two group parameters, Kruskal-Wallis for multiple groups and the Chi-square test in cases of dichotomous response parameters and to test differences in proportions between groups. Multivariate analyses were used to explore the magnitude of differences in DFS with and without second primary other cancer as a survival endpoint. Variables that often have been shown to be of prognostic significance in CRC were selected for the model [13-15]. Hazard ratio (HR), with 95% confidence intervals (CI), was calculated by a Cox proportional hazards model. All P values were two-sided, and statistically significant differences were assumed when p < 0.05.

## Results

The median (range) follow-up time for surviving patients was 8 (6-10) years. The median age of the cohort (n = 415) was 73 (34-97) years, males represented 203 (49%) patients and the disease location was colon in 284 (68%) patients and rectum in 131 (32%). The distribution of disease stage (TNM) was 57 (14%) stage I; 150 (36%) stage II; 125 (30%) stage III; 68 (16%) stage IV and 15 (4%) unknown. The median number of lymph nodes analysed was 15 (0-55) and a 30 day mortality was seen in 13 (3%) patients.

Table 2 presents counts of events in patients treated with curative intention during a follow-up time of eight years. The most frequent events were non-cancer related death, distant metastases and death from CRC. An intermediate group of common events comprised second primary other cancers, and rare events were locoregional recurrence, second primary CRC, death from other cancer, treatment-related death defined as postoperative mortality within 30 days and loss to follow-up (Table 2). DFS was the survival endpoint with the largest number of events and CSS with the fewest. Most OS and DFS events occurred during the first three years (Table 2).

**Table 2:** Counts of events in patients with colorectal cancer treated with curative intention (n = 332).

Years from diagnosis	1	2	3	4	5	6	7	8	
Number entering the interval	332	305	286	261	242	221	209	156	

**Type of event in numbers**									Total

Locoregional recurrence	1	2	0	0	1	0	0	0	4

Distant metastases	13	20	13	10	6	2	2	0	66

Second primary, same cancer	0	2	1	1	0	0	0	0	4

Second primary, other cancer	2	4	6	5	4	3	1	1	26

Death from same cancer	6	10	13	12	8	3	6	1	59

Death from other cancer	1*	0	1*	1	0	2	0	0	5

Non - cancer-related death	14	9	11	6	12	7	7	8	74

Treatment-related death	6	0	0	0	0	0	0		6

Loss to follow-up	0	0	0	1	0	0	0		1

**Number of events for each type of survival endpoint**									

DFS	36	35	29	18	22	12	10	8	170

RFS	34	29	25	16	18	9	4	4	139

OS	27	19	25	19	20	12	13	9	144

TTF	20	22	15	11	6	4	2	0	80

TTR	14	20	13	10	7	2	2	0	68

CSS	6	10	13	12	8	3	6	1	59

**Cumulative number of events at 3 and 5 years n**									

DFS			100		140				155

OS			71		110				125

Numbers are presented up to 8 years of follow-up.

* Death from other cancer diagnosed before the primary CRC. DFS: disease-free survival; RFS: relapse-free survival; TTF: time to treatment failure; TTR: time to recurrence; CSS: cancer-specific survival; OS: overall survival

**Figure 1:**
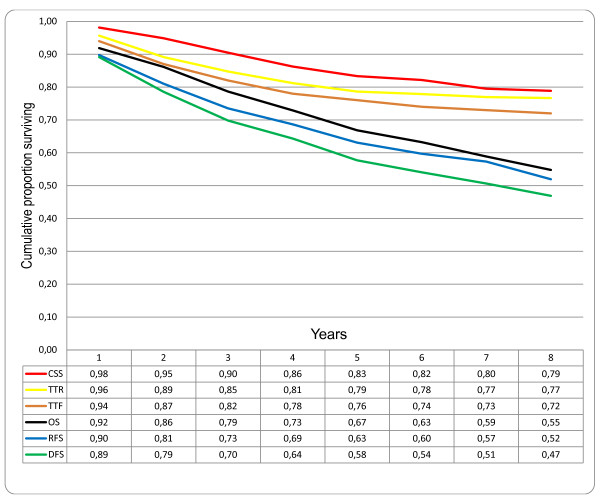
**Comparison of different survival endpoints in patients treated with curative intention for colorectal cancer, disease stages I-III (n = 332)**. CSS: cancer specific survival; TTR: time to recurrence; TTF = time to treatment failure; OS: overall survival; RFS: relapse-free survival; DFS: disease-free survival.

When comparing the survival curves of different types of endpoint in patients treated with curative intention it was observed that TTR initially has a steeper slope than CSS, but that the curves merge with time (Figure 1). Similar observations were seen with RFS and OS (Figure 1).

**Figure 2:**
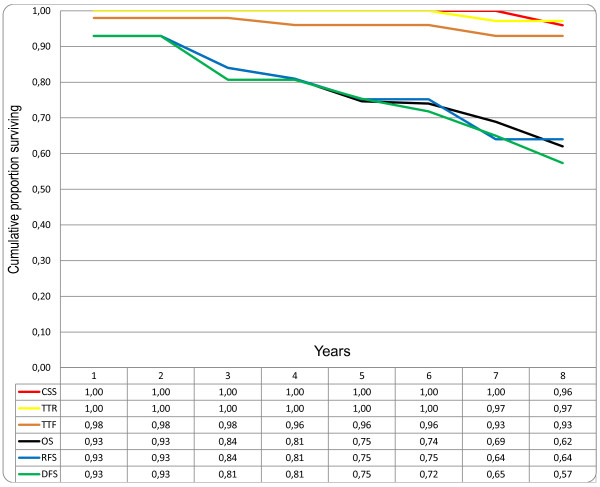
**Comparison of different survival endpoints in patients treated with curative intention for colorectal cancer, disease stage I (n = 57)**. CSS: cancer specific survival; TTR: time to recurrence; TTF = time to treatment failure; OS: overall survival; RFS: relapse-free survival; DFS: disease-free survival.

**Figure 3:**
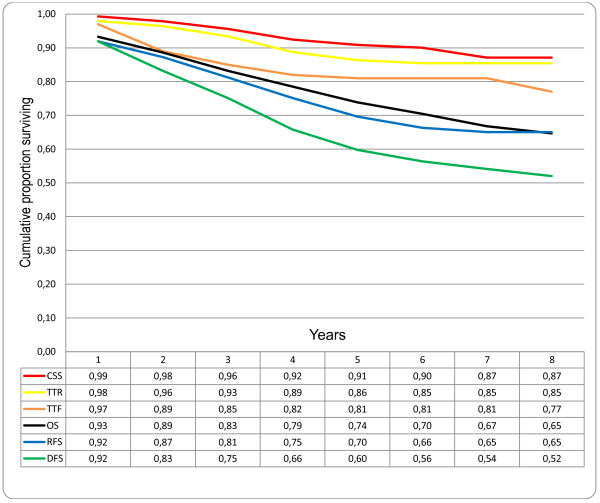
**Comparison of different survival endpoints in patients treated with curative intention for colorectal cancer, disease stage II (n = 149)**. CSS: cancer specific survival; TTR: time to recurrence; TTF = time to treatment failure; OS: overall survival; RFS: relapse-free survival; DFS: disease-free survival.

**Figure 4:**
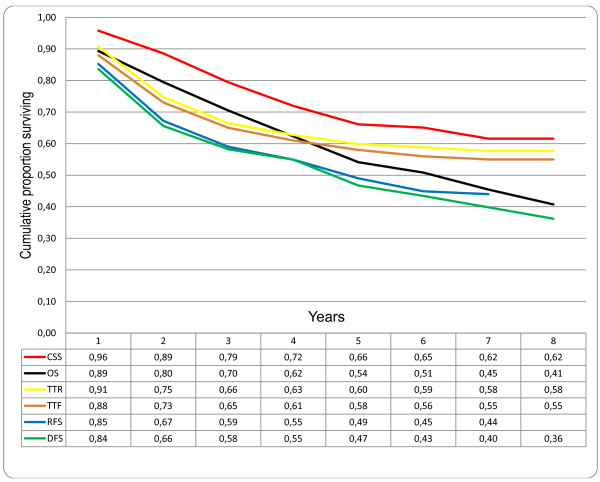
**Comparison of different survival endpoints in patients treated with curative intention for colorectal cancer, disease stage III (n = 122)**. CSS: cancer specific survival; TTR: time to recurrence; TTF = time to treatment failure; OS: overall survival; RFS: relapse-free survival; DFS: disease-free survival.

When analysing the same endpoints in individual disease stages, the curves for TTR and CSS have similar patterns for both stages I and II due to few recurrences in stage I (Figure 2) and to high mortality from CRC recurrence in stage II (Figure 3). For stage III the curves for TTR and CSS continue to be separated by more events in TTR during the first five years, representing patients surviving after CRC recurrence (Figure 4). An additional observation is that proportionally more patients with stage II develop late recurrences after three years compared with stage III patients. In stage II, 11 (55%) out of 20 recurrences occurred after three years of follow-up compared with 8 (17%) out of 47 recurrences in stage III patients (P = 0.002).

When continuing the comparison between stages II and III, a different progression of the survival curves for OS and DFS between three and five years is observed; for stage II the curves diverge from an 8 to a 14% difference and the curves merge for stage III from 12 to 7% (Figure 3 and 4). This observation is explained by a trend for more second primary other cancers in patients with stage II (12%) compared with stage III (6%) after five years of follow-up (P = 0.073). The differences in the cumulative proportion of patients without second primary other cancers between stages II and III are greatest during three to five years of follow-up. The cumulative proportions, with standard error, of patients without second primary other cancer after three years were 92.5 ± 2.5 for stage II and 98.2 ± 1.2 for stage III and after 5 years 87.5 ± 2.9 for stage II and 92.9 ± 2.9 for stage III.

**Figure 5:**
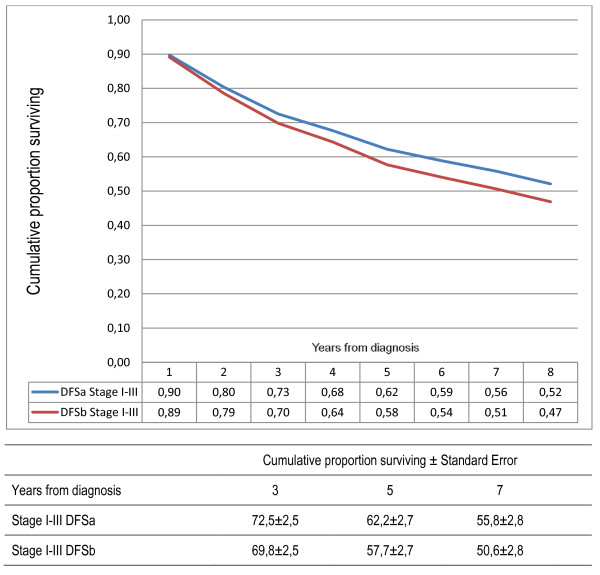
**Comparison of two different definitions of disease-free survival (DFS) in patients curatively treated for colorectal cancer stages I-III (n = 332)**. For DFSa only second primary same cancer was included as an event and second primary other cancer was ignored. For DFSb all second primary cancers were counted as an event. The cumulative proportion with standard deviations at 3, 5 and 7 years are given.

**Figure 6:**
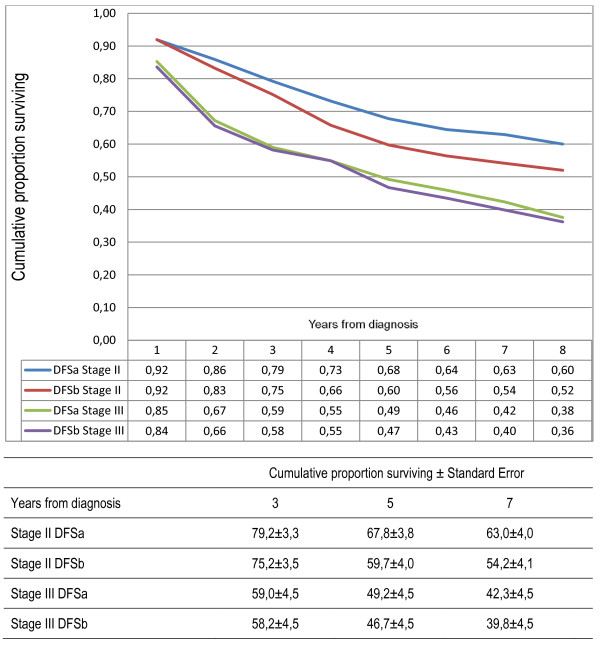
**Comparison of two different definitions of disease-free survival (DFS) in patients curatively treated for colorectal cancer stage stage II (n = 149) and III (n = 122)**. For DFSa only second primary same cancer was included as an event and second primary other cancer was ignored. For DFSb all second primary cancers were counted as an event. The cumulative proportion with standard deviations at 3, 5 and 7 years are given.

Furthermore, when two different definitions of DFS were compared, one including only second primary CRC and the other including all second primary cancers, a worse DFS was observed if second primary other cancers were included. DFS for patients with stage I-III disease was 62% after five years if second primary other cancer was not included as an event, compared with 58% if second primary other cancer was included (Figure 5). However, the difference was larger for stage II (68 vs 60%) than for stage III (49 vs 47%)(Figure 6). In multivariate analyses, emergency operation became a statistically significant parameter of poor prognosis if second primary other cancers were included as an event in DFS (Table 3).

**Table 3:** Multivariate Cox proportional hazard models of disease-free survival (DFS) in 332 patients with colorectal cancer stages I-III treated with curative intention.

	DFSa			DFSb		
	**HR**	**95.0% CI**		**HR**	**95.0% CI**	

		**Lower**	**Upper**		**Lower**	**Upper**

Female	1.00			1.00		

Male	0.85	0.62	0.18	0.90	0.66	1.22

Age ≤ 75 years	1.00			1.00		

Age > 75 years	2.68	1.92	3.73	2.50	1.83	3.42

Elective operation	1.00			1.00		

Emergency operation	1.64	0.94	2.86	2.06	1.23	3.46

Colon	1.00			1.00		

Rectum	1.20	0.83	1.73	1.17	0.82	1.66

< 12 lymph nodes analysed	1.00			1.00		

≥ 12 lymph nodes analysed	1.13	0.79	1.60	1.11	0.79	1.55

Stage I	1.00			1.00		

Stage II	1.23	0.72	2.11	1.43	0.86	2.36

Stage III	2.52	1.49	4.27	2.25	1.35	3.74

Well-moderate tumour differentiation	1.00			1.00		

Poor tumour differentiation	1.02	0.69	1.53	1.18	0.82	1.71

No vascular invasion	1.00			1.00		

Vascular invasion	1.44	0.85	2.44	1.50	0.91	2.48

For DFSa only second primary same cancer was included as an event and second primary other cancer was ignored. For DFSb all second primary cancers were counted as an event.

The differences between stages II and III result from more CRC deaths and fewer second primary other cancers in patients with stage III (n = 7) compared to patients with stage II disease (n = 18). Eleven (8%) out of 142 patients receiving pre- or postoperative (neo-) adjuvant treatment developed a second primary other cancer compared to 17 (9%) out of 188 patients not receiving this treatment. The most common types of second primary other cancers were breast (n = 7), lung (n = 3) and prostate (n = 3) cancer with no differences in type between stages. Stage III patients were younger (P = 0.037) compared with stage II patients. For 283 patients information on heredity was available. No differences were seen between stages in the proportion of patients with first degree relatives with CRC, numbering for stage II 12 (10%) out of 122 and for stage III 9 (10%) out of 91. No differences were seen in the age at diagnosis for patients with and without second primary other cancer.

## Discussion

The present study reveals that inclusion of second primary other cancers affects the results of DFS in patients with CRC. Second primary other cancers generate a significant number of events during follow-up of patients with CRC, which causes worse DFS when the second primary other cancers are included as an event in the calculations. Almost half as many second primary other cancers were observed as endpoints as death from the same cancer or non-cancer-related deaths. The most common events to occur were distant metastases closely followed by death from CRC and non-cancer related deaths, while second primary CRC was rarely seen.

The inclusion of second primary other cancer as an event in DFS did also influence the results of multivariate Cox models, in our example causing significant changes in the HR for emergency operation. Interestingly, the inclusion of second primary other cancers had a more detrimental effect on DFS of stage II patients than stage III patients, and this effect became greater with increasing time from diagnosis. The observation of more second primary other cancers in stage II patients than other disease stages has been reported by others [16]. The reason for this is not known. In the present study second primary other cancers were more common in those with first degree relatives with CRC, however, the proportion was similar in stages II and III, and no association was seen with adjuvant treatment. This indicates that the second other cancers are likely not therapy-induced. The reason for the greater effect of second primary other cancer on DFS survival in stage II is therefore largely explained by fewer events from other endpoints such as distant metastasis and death from CRC.

Second primary other cancers are significantly more common in patients with CRC than in the normal population [17,18]. One reason for this can be related to radiation therapy for rectal cancer [19], although this theory has been debated [20]. Patients with family history of CRC are also more likely to develop second primary other cancers than patients without heredity [10,21].

When studying the efficacy of adjuvant cancer treatments, the primary aim is to see whether the treatment reduces the risk of recurrence and subsequent death in the cancer of interest. In this situation TTR and CSS are the most specific endpoints because they only depend on events directly related to CRC and the effect of age is small. However, it is important to determine whether the investigated treatment is safe or whether it has serious adverse effects. Analyses that include events such as treatment-related death, non-cancer related death and second primary other cancer may then be necessary, thus OS and DFS, which include these events, serve as the primary endpoints in most randomised adjuvant trials.

Three year DFS has been suggested as a surrogate endpoint for five year OS in the setting of adjuvant treatment CRC trials [5]. In the study second primary other cancer was not included as an endpoint in DFS [5]. A recent publication has suggested that three year DFS is superior to five year OS and comparable with six and seven year OS, since extended survival due to more effective cancer treatments after disease recurrence is frequently seen [22]. Unfortunately it is not possible to determine whether or not second primary other cancers are included as a DFS endpoint in these analyses. This is important as after three years second primary other cancers already generate a significant number of events that can affect the results of the DFS.

It is essential that endpoints in studies of CRC are clearly defined as this will increase the comparability of studies. The events selected should be simple to collect to minimise the risk of missing events, and the definitions should be pre-specified at the time of study design. An example of a recent study lacking definition of the endpoints is the study on the prognostic value of KRAS and BRAF [23]. RFS and OS were analyzed according to stage in patients included in three adjuvant chemotherapy trials. The study is the largest of its type and published in a high impact journal and should therefore serve as a reliable reference for others, but it is not known if RFS refers to time to recurrence only or time to recurrence and death, nor is it clear if second primary other cancer is included as an event. This makes it difficult to interpret the results and it is impossible to compare the RFS to other studies.

A good example of endpoint definitions is found in a meta-analysis of 18 adjuvant treatment trials in colon cancer [24]. In this study OS, DFS and TTR were clearly defined: "OS is defined as time to death from any cause. DFS is defined as the time to recurrence or death, whichever occurs first. TTR is defined as the time to disease recurrence, where deaths without recurrence were censored at the time of death. Recurrence was defined only by a reappearance of primary colon cancer; second primary colon cancers or other non-colon cancers were not classified as recurrences."

The present study is a population-based observational study, with some of the endpoints retrospectively collected. This results in an older population with a broader range of stage at diagnosis and less reliable information on disease recurrence, secondary cancers and causes of death than an adjuvant treatment trial. Yet a population-based cohort, as in the present study, has the advantage of limited patient selection, frequently being very large in clinical trials [25]. However, our study setting represents a real life situation and a population that would be the recruitment base for clinical studies. Furthermore, the proportion of patients with second other cancers is, in CRC, largely independent of age and disease stage. Therefore we could expect an at least similar effect of second primary other cancers on DFS survival calculations in an adjuvant treatment trial compared with the present study.

It is debatable whether second primary other cancer should be regarded as a primary endpoint or as an adverse effect and therefore not included as an event in the main analysis of DFS. To increase clarity Punt [4] recommends that if second primary other cancers are ignored as an event, the survival endpoint should be named RFS. Choice of survival endpoints is an important topic and, to the best of our knowledge, this is the first study to address the use of second primary other cancers in DFS calculations in CRC.

## Conclusions

Different definitions of survival endpoints have a significant effect on the survival analyses. Inclusion of second primary other cancers as an endpoint in DFS analysis significantly alters the survival for patients with CRC. Researchers and journals must clearly define survival endpoints in all trial protocols and published manuscripts. To minimise the differences in survival calculations between studies and to enable more precise comparisons of studies we recommend the general use of the definition of endpoints published in the consensus document of Punt et al [4].

## Competing interests

The authors declare that they have no competing interests.

## Authors' contributions

HB collected the clinical information and performed the statistical analysis. All authors participated in the design, data interpretation, manuscript editing and review. All authors read and approved the final manuscript.

## Pre-publication history

The pre-publication history for this paper can be accessed here:

http://www.biomedcentral.com/1471-2407/11/438/prepub

## References

[B1] Altman DG Schulz KF Moher D Egger M Davidoff F Elbourne D Gotzsche PC Lang T The revised CONSORT statement for reporting randomized trials: explanation and elaboration Ann Intern Med 2001 134 8 663 694 11304107 10.7326/0003-4819-134-8-200104170-00012

[B2] Moher D Schulz KF Altman D The CONSORT statement: revised recommendations for improving the quality of reports of parallel-group randomized trials Jama 2001 285 15 1987 1991 10.1001/jama.285.15.1987 11308435

[B3] Mathoulin-Pelissier S Gourgou-Bourgade S Bonnetain F Kramar A Survival end point reporting in randomized cancer clinical trials: a review of major journals J Clin Oncol 2008 26 22 3721 3726 10.1200/JCO.2007.14.1192 18669458

[B4] Punt CJ Buyse M Kohne CH Hohenberger P Labianca R Schmoll HJ Pahlman L Sobrero A Douillard JY Endpoints in adjuvant treatment trials: a systematic review of the literature in colon cancer and proposed definitions for future trials J Natl Cancer Inst 2007 99 13 998 1003 10.1093/jnci/djm024 17596575

[B5] Sargent DJ Wieand HS Haller DG Gray R Benedetti JK Buyse M Labianca R Seitz JF O'Callaghan CJ Francini G Grothey A O'Connell M Catalano PJ Blanke CD Kerr D Green E Wolmark N Andre T Goldberg RM De Gramont A Disease-free survival versus overall survival as a primary end point for adjuvant colon cancer studies: individual patient data from 20,898 patients on 18 randomized trials J Clin Oncol 2005 23 34 8664 8670 10.1200/JCO.2005.01.6071 16260700

[B6] Chua YJ Sargent D Cunningham D Definition of disease-free survival: this is my truth-show me yours Ann Oncol 2005 16 11 1719 1721 10.1093/annonc/mdi373 16192295

[B7] Barlow L Westergren K Holmberg L Talback M The completeness of the Swedish Cancer Register: a sample survey for year 1998 Acta Oncol 2009 48 1 27 33 10.1080/02841860802247664 18767000

[B8] Pahlman L Bohe M Cedermark B Dahlberg M Lindmark G Sjodahl R Ojerskog B Damber L Johansson R The Swedish rectal cancer registry Br J Surg 2007 94 10 1285 1292 10.1002/bjs.5679 17661309

[B9] Birgisson H Nielsen HJ Christensen IJ Glimelius B Brunner N Preoperative plasma TIMP-1 is an independent prognostic indicator in patients with primary colorectal cancer: a prospective validation study Eur J Cancer 2010 46 18 3323 3331 10.1016/j.ejca.2010.06.009 20619633

[B10] Birgisson H Ghanipour A Smedh K Pahlman L Glimelius B The correlation between a family history of colorectal cancer and survival of patients with colorectal cancer Fam Cancer 2009 8 4 555 561 10.1007/s10689-009-9286-0 19714489

[B11] Quality registers in Sweden http://www.kvalitetsregister.se/om_kvalitetsregister/quality_registries

[B12] von Elm E Altman DG Egger M Pocock SJ Gotzsche PC Vandenbroucke JP The Strengthening the Reporting of Observational Studies in Epidemiology (STROBE) statement: guidelines for reporting observational studies J Clin Epidemiol 2008 61 4 344 349 10.1016/j.jclinepi.2007.11.008 18313558

[B13] Compton CC Fielding LP Burgart LJ Conley B Cooper HS Hamilton SR Hammond ME Henson DE Hutter RV Nagle RB Nielsen ML Sargent DJ Taylor CR Welton M Willett C Prognostic factors in colorectal cancer. College of American Pathologists Consensus Statement 1999 Arch Pathol Lab Med 2000 124 7 979 994 10888773 10.5858/2000-124-0979-PFICC

[B14] Figueredo A Coombes ME Mukherjee S Adjuvant therapy for completely resected stage II colon cancer Cochrane Database Syst Rev 2008 3 CD005390 10.1002/14651858.CD005390.pub2 PMC8885310 18646127

[B15] Zlobec I Lugli A Prognostic and predictive factors in colorectal cancer J Clin Pathol 2008 61 5 561 569 18326017 10.1136/jcp.2007.054858

[B16] Chiang JM Yeh CY Changehien CR Chen JS Tang R Tsai WS Fan CW Clinical features of second other-site primary cancers among sporadic colorectal cancer patients--a hospital-based study of 3,722 cases Hepatogastroenterology 2004 51 59 1341 1344 15362748

[B17] Noura S Ohue M Seki Y Tanaka K Motoori M Kishi K Miyashiro I Ohigashi H Yano M Ishikawa O Tsukuma H Murata K Kameyama M Second Primary Cancer in Patients with Colorectal Cancer after a Curative Resection Dig Surg 2009 26 5 400 405 10.1159/000229991 19923828

[B18] Evans HS Moller H Robinson D Lewis CM Bell CM Hodgson SV The risk of subsequent primary cancers after colorectal cancer in southeast England Gut 2002 50 5 647 652 10.1136/gut.50.5.647 11950810 PMC1773208

[B19] Birgisson H Pahlman L Gunnarsson U Glimelius B Occurrence of second cancers in patients treated with radiotherapy for rectal cancer J Clin Oncol 2005 23 25 6126 6131 10.1200/JCO.2005.02.543 16135478

[B20] Kendal WS Nicholas G A population-based analysis of second primary cancers after irradiation for rectal cancer Am J Clin Oncol 2007 30 4 333 339 10.1097/01.coc.0000258084.55036.9e 17762431

[B21] Hemminki K Li X Dong C Second primary cancers after sporadic and familial colorectal cancer Cancer Epidemiol Biomarkers Prev 2001 10 7 793 798 11440965

[B22] de Gramont A Hubbard J Shi Q O'Connell MJ Buyse M Benedetti J Bot B O'Callaghan C Yothers G Goldberg RM Blanke CD Benson A Deng Q Alberts SR Andre T Wolmark N Grothey A Sargent D Association between disease-free survival and overall survival when survival is prolonged after recurrence in patients receiving cytotoxic adjuvant therapy for colon cancer: simulations based on the 20,800 patient ACCENT data set J Clin Oncol 2010 28 3 460 465 10.1200/JCO.2009.23.1407 20008641 PMC2815708

[B23] Roth AD Tejpar S Delorenzi M Yan P Fiocca R Klingbiel D Dietrich D Biesmans B Bodoky G Barone C Aranda E Nordlinger B Cisar L Labianca R Cunningham D Van Cutsem E Bosman F Prognostic role of KRAS and BRAF in stage II and III resected colon cancer: results of the translational study on the PETACC-3, EORTC 40993, SAKK 60-00 trial J Clin Oncol 28 3 466 474 10.1200/JCO.2009.23.3452 20008640

[B24] Sargent D Sobrero A Grothey A O'Connell MJ Buyse M Andre T Zheng Y Green E Labianca R O'Callaghan C Seitz JF Francini G Haller D Yothers G Goldberg R de Gramont A Evidence for cure by adjuvant therapy in colon cancer: observations based on individual patient data from 20,898 patients on 18 randomized trials J Clin Oncol 2009 27 6 872 877 10.1200/JCO.2008.19.5362 19124803 PMC2738431

[B25] Sorbye H Pfeiffer P Cavalli-Bjorkman N Qvortrup C Holsen MH Wentzel-Larsen T Glimelius B Clinical trial enrollment, patient characteristics, and survival differences in prospectively registered metastatic colorectal cancer patients Cancer 2009 115 20 4679 4687 10.1002/cncr.24527 19562777

